# The Incidence of Tetrodotoxin and Its Analogs in the Indian Ocean and the Red Sea

**DOI:** 10.3390/md17010028

**Published:** 2019-01-05

**Authors:** Isidro José Tamele, Marisa Silva, Vitor Vasconcelos

**Affiliations:** 1 CIIMAR/CIMAR—Interdisciplinary Center of Marine and Environmental Research, University of Porto, Terminal de Cruzeiros do Porto, Avenida General Norton de Matos, 4450-238 Matosinhos, Portugal; isitamele@gmail.com (I.J.T.); marisasilva17@gmail.com (M.S.); 2 Institute of Biomedical Science Abel Salazar, University of Porto, R. Jorge de Viterbo Ferreira 228, 4050-313 Porto, Portugal; 3 Faculty of Sciences, Eduardo Mondlane University, Av. Julius Nyerere, nr 3453, Campus Principal, 257 Maputo, Mozambique; 4 Department of Biology, Faculty of Sciences, University of Porto, Rua do Campo Alegre, 4619-007 Porto, Portugal

**Keywords:** Indian Ocean, Red Sea, tetrodotoxin, pufferfish poisoning

## Abstract

Tetrodotoxin (TTX) is a potent marine neurotoxin with bacterial origin. To date, around 28 analogs of TTX are known, but only 12 were detected in marine organisms, namely TTX, 11-oxoTTX, 11-deoxyTTX, 11-norTTX-6(R)-ol, 11-norTTX-6(S)-ol, 4-*epi*TTX, 4,9-anhydroTTX, 5,6,11-trideoxyTTX, 4-CysTTX, 5-deoxyTTX, 5,11-dideoxyTTX, and 6,11-dideoxyTTX. TTX and its derivatives are involved in many cases of seafood poisoning in many parts of the world due to their occurrence in different marine species of human consumption such as fish, gastropods, and bivalves. Currently, this neurotoxin group is not monitored in many parts of the world including in the Indian Ocean area, even with reported outbreaks of seafood poisoning involving puffer fish, which is one of the principal TTX vectors know since Egyptian times. Thus, the main objective of this review was to assess the incidence of TTXs in seafood and associated seafood poisonings in the Indian Ocean and the Red Sea. Most reported data in this geographical area are associated with seafood poisoning caused by different species of puffer fish through the recognition of TTX poisoning symptoms and not by TTX detection techniques. This scenario shows the need of data regarding TTX prevalence, geographical distribution, and its vectors in this area to better assess human health risk and build effective monitoring programs to protect the health of consumers in Indian Ocean area.

## 1. Introduction

The tropical and subtropical climates predominant in the Indian Ocean zone, accompanied by industrialization and population increase, are pointed to as the main factors that, together with eutrophication, contribute to the development of toxic phytoplankton blooms—harmful algal blooms (HABs) and bacteria [1]. HABs and some bacteria are marine toxin (MT) producers, turning the Indian Ocean zone vulnerable to this phenomenon [2,3,4,5]. One of the main Indian Ocean MTs is tetrodotoxin (a neurotoxin) and its analogs (TTXs), of which the main producers were reported to belong to different bacteria genera [6,7,8,9,10,11,12,13,14,15]. Cases of human poisoning are recurrent, especially after consumption of TTX-contaminated fish, with the puffer fish as the most common vector reported since Egyptian times [16,17,18,19,20,21,22,23,24,25,26,27,28,29]. Due to the lack of TTX monitoring programs, the episodes of human seafood poisoning are still common in the Indian Ocean area, since seafood is the most common food for many people living along coastal zones [16,17,18,19,20,21,22,24,26,28,29,30,31,32,33,34,35,36,37,38]. Thus, the objective of this paper was to review the incidence of TTX in the Indian Ocean and the Red Sea zones and associated human seafood poisoning incidents. The monitoring of TTXs in this geographic zone is also recommended.

## 2. Tetrodotoxin

**Figure 1:**
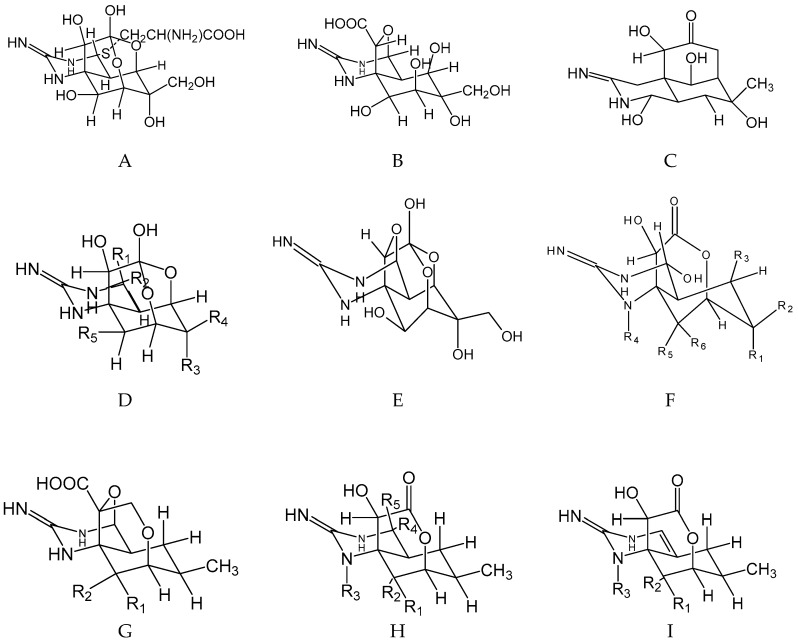
Tetrodotoxin (TTX) and analogs modified from European Food Safety Authority (EFSA) 2017 [45] and Yotsu-Yamasshita et al. (2007) [15,53,54]. (*) indicates TTX analogs that occur in marine organisms with known relative toxicity. (**A**) 4-cysTTX(*), (**B**) tetrodonic acid, (**C**) 4,9-anhydroTTX(*), (**D**) 1-hydroxy-5,11-dideoxyTTX, (**E**) TTX and 12 analogs, (**F**) 5-deoxyTTX(*) and three analogs, (**G**) trideoxyTTX and two analogs, (**H**) 4-epi-5,6,11-trideoxyTTX and another analog, and (**I**) 4,4a-anhydro-5,6,11-trideoxyTTX and 1-hydroy-4,4a-anhydro-8-epi-5,5,11-trideooxyTTX (see radicals of the analogs in the Table 1).

TTX (Figure 1) is a potent neurotoxin group [39] that can provoke severe poisoning after consumption of contaminated seafood. Several species of distinct marine organisms of human consumption were identified as TTX vectors: puffer fish [16,17,18,19,20,21,22,23,24,25,26,27,28,29], gastropods [40], crustaceans [41,42,43,44], and bivalves [45]. Also, the occurrence of TTXs in terrestrial vertebrates such as *Polypedates* sp., *Atelopus* sp., *Taricha granulosa*, [46] and *Cynops ensicauda popei* [47] was reported [48,49]. TTX is an alkaloid isolated for the first time in 1909 by Tahara and Hirata from the ovaries of globefish [50]. In the marine environment, bacteria are pointed to as the main producers of this group of toxins, namely *Serratia marcescens* [51], *Vibrio alginolyticus*, *V. parahaemolyticus, Aeromonas* sp. [52], *Microbacterium arabinogalactanolyticum* [13], *Pseudomonas* sp. [14], *Shewanella putrefaciens* [6], *Alteromonas* sp. [8], *Pseudoalteromonas* sp. [10], and *Nocardiopsis dassonvillei* [12]. Physicochemically, TTXs are colorless, crystalline weak heterocyclic basic compounds (Figure 1 and Table 1), highly hydro-soluble and also heat-stable [45]; thus, the toxin is not destroyed by cooking procedures.

To date, around 28 analogs of TTX were described (Figure 1 and Table 1) and some of them were detected in marine organisms [53], with their relative toxicity well known [45] (chemical structures pointed with asterisks in Figure 1): TTX, 11-oxoTTX, 11-deoxyTTX, 11-norTTX-6(R)-ol, 11-norTTX-6(S)-ol, 4-*epi*TTX, 4,9-anhydroTTX, 5,6,11-trideoxyTTX [45], 4-CysTTX, 5-deoxyTTX, 5,11-dideoxyTTX, and 6,11-dideoxyTTX [54,55,56,57] (Table 1). Their relative toxicity ranges from 0.01 to 1.0, with 5,6,11-trideoxyTTX and TTX as the least and most toxic, respectively [45], and there are still no available data regarding the toxicity for 4-CysTTX and 5,11-dideoxyTTX. Chemical abstract numbers (CAS) are also listed in Table 2.

The action mechanism of TTXs occurs through the occlusion of the external pore of site 1 of voltage-gated sodium channels on the surface of nerve membranes, blocking cellular communication and causing death by cardio-respiratory paralysis [60]. Paralysis occurs by affecting the respiratory system, the diaphragm, skeletal muscles, and tissues in the digestive tract in humans [39]. TTXs normally accumulate in skin, intestines, liver, muscle, gonads, viscera, and ovaries in different species of puffer fish [16,21,22,29,33,34,35,36,37,61,62,63,64,65]. The symptoms that can be used partially as an indication of TTX human poisoning (wt = 50 kg and TTX amount = 2 mg) were grouped into four levels depending on the amount ingested [66] and are described in Table 3. These symptoms normally appear 40 min after consumption of contaminated food and, in some cases, even six hours after [67].

Currently, there is no antidote for TTX; however, some studies indicate that the application of activated charcoal could help in reversing the clinical stage of poisoning victims since it reduces the toxin free amount [68]. Also, alkaline gastric lavage with sodium bicarbonate (2%) is indicated as a treatment within the first hour of the incident, due to TTX instability in alkaline media [69]. Another clinical intervention recommendation is the use of cholinesterase inhibitors such as neostigmine [28], and mechanical respiratory help may reduce mortality probability by muscle paralysis [38].

## 3. TTX Detection Methods

Several methodologies were developed to analyze TTXs and, in recent years, chemical methods became more popular due to their sensitivity with limits of detection (LODs) ranging from 0.9 ng to 0.063 μg. Liquid chromatography with tandem mass spectrometry (LC–MS/MS) techniques, the first choice compared to mouse bioassays (MBAs) and enzymatic methods due to their greater sensitivity and specificity, have the capacity to detect and determine TTXs in complex matrices [70]. Also, due to ethical reasons and lack of specificity, MBA fell into disuse, with the latter reason also attributed to the enzymatic methods. When a poisoning case occurs, it is recommended, when available, to screen the liver, muscle, skin, gonads, and ovaries of the suspected poisoning marine vector samples [28,36,40,41,42,53,54,55,56,62,70,71,72,73,74,75,76,77,78,79,80,81,82,83,84,85,86,87,88]. Human urine and plasma should also be analyzed for TTX in these cases [80]. 

Methods for TTX analysis and their respective limits of quantification (LOQs) and detection (LODs) are described in Table 4 and include the mouse bioassay [12,36,52,89], receptor-based assay [90], immunoassay [31,36,52,73,77,82,89,91,92,93], thin-layer chromatography [13,72], high-performance liquid chromatography [84,94,95], gas chromatography–mass spectrometry [76,84,95], liquid chromatography coupled to mass spectrometry [33,40,96,97,98], surface plasmon resonance [30], and liquid chromatography with fluorescence detection (FLD) [15,32,89]. 

## 4. Geographic Occurrence and Incidence of TTXs in the Indian Ocean and the Red Sea

As described in the introduction section, TTXs were reported in several marine organisms [71], regarding poisoning incidents [71]; the main TTX vectors involved in the Indian Ocean and the Red Sea (Table 4) belong to the Tetraodontidae family: *Arothron hispidus* in India [65], *Takifugu oblongus* in Bangladesh [16,33] and India [35,62], *Lageocephalus scitalleratus* in Singapure [20], *Pleuranacanthus sceleratus* in Egypt [21,34,37], Reunion Island [29], and Australia [23,24], *Chelonodon pataca, Sphaeroides oblongus*, *Lagocephalus inermis*, and *Lagocephalus lunaris* in India [35,62], *Xenopterus naritus* in Malaysia [63], *Arothron stellatus* in India [64], *Tetractenos hamiltoni* in Australia [80,100], and *Tetroadon* sp. [17], *Tetraodon nigroviridis,* and *Arothron reticularis* in Thailand [99]. The records of TTX occurrence in other marine species such as mollusks are scarce in the Indian Ocean. Gastropods were reported as TTX vectors in other locations: *Charonia lampas* [85], *Gibbula umbilicalis,* and *Monodonta lineata* on the Portuguese coast [40], *Nassarius* spp. in China [94], *Polinices didyma*, *Natica lineata* [84,101], *Oliva miniacea*, *O. mustelina*, and *O. nirasei* [95] in Taiwan, *Charonia sauliae* [102], *Babylonia japonica* [86], *Niotha* spp. [75,81], and *Tutufa lissostoma* [103] in Japanese crabs, *Demania cultripes, Demania toxica, Demania reynaudi, Lophozozymus incises*, *Lophozozymus pictor*, *Atergatis floridus* [104], and *Atergatopsis germaini* [83], highlightinh these organisms as potential indicator species [11]. Data on these groups are scarce in the Indian Ocean area, suggesting that further studies and monitoring programs for TTXs are needed. Available data regarding this geographic region are displayed in Table 5.

## 5. Final Considerations

TTX data in the Indian Ocean and Red Sea are usually related to fatal outbreaks due to seafood poisoning and not to scientific research, indicating the lack of MT monitoring programs. The symptomatology reports and MBA are used to identify seafood poisoning caused by TTX and analogs, indicating the need for analytical methods such as liquid chromatography to obtain better quantitative data. Both symptomatology and MBA in isolation are not enough to conclude that TTXs are the causative agent of seafood poisoning, since there are other toxins (PSTs) with similar action mechanism that overlap in symptomatology with TTX poisoning. Additionally, MBA cannot discriminate between the different TTX analogs. MBA and symptomatology are used in countries of the Indian Ocean and the Red Sea to identify TTX poisoning due to the lack of availability and accessibility to chemical methods and the absence of TTX monitoring programs. 

Thus, the implementation of monitoring programs using chemical analytical methods such as LC–MS/MS instead of MBA in the Indian Ocean and the Red Sea is urgently needed in different species of shellfish and puffer fish, including *Arothron hispidus*, *Takifugu oblongus*, *Lageocephalus scitalleratus*, *Pleuranacanthus sceleratus*, *Chelonodon patoca*, *Sphaeroides oblongus*, *Lagocephalus inermis*, *Lagocephalus lunaris*, *Xenopterus naritus*, *Arothron stellatus*, *Tetractenos hamiltoni*, *Tetraodon nigroviridis*, *Arothron reticularisand*, *Charonia sauliae*, *Babylonia japonica*, *Niotha* spp., and *Tutufa lissostoma*, since they are most consumed and are already confirmed to be vectors of TTX in the Indian Ocean and the Red Sea. These species can be used as indicators for monitoring programs using the maximum limit permitted of 2 mg·kg^−1^ (from Japan).

## Figures and Tables

**Table 1:** Tetrodotoxin (TTX) and analogs shown in Figure 1 and modified from European Food Safety Authority (EFSA) 2017 [45] and Yotsu-Yamasshita et al. (2007) [15,53].

**E**	**R1**	**R2**	**R3**	**R4**	**R5**	
TTX (*)	H	OH	OH	CH_2_OH	OH	
4-*epi*TTX (*)	OH	H	OH	CH_2_OH	OH	
6-epiTTX (*)	H	OH	CH_2_OH	OH	OH	
11-deoxyTTX (*)	H	OH	OH	CH3	OH	
6,11-dideoxyTTX	H	OH	H	CH3	OH	
8,11-dideoxyTTX	H	OH	OH	CH3	H	
11-oxoTTX (*)	H	OH	OH	CH(OH)_2_	OH	
11-norTTX-6,6-diol	H	OH	OH	OH	OH	
11-norTTX-6(R)-ol (*)	H	OH	H	OH	OH	
11-norTTX-6(S)-ol (*)	H	OH	OH	H	OH	
Chiriquitoxin	H	OH	OH	CH(OH)CH(NH_3_^+^)COO^−^	OH	
TTX-8-*O*-hemisuccinate	H	OH	OH	CH_2_OH	OOC(CH_2_)_2_COO^−^	
TTX-11-carboxylic acid	H	OH	OH	COO^−^	OH	
TTX (*)	H	OH	OH	CH_2_OH	OH	
**F**	**R1**	**R2**	**R3**	**R4**	**R5**	**R6**
5-deoxyTTX(*)	OH	CH_2_OH	H	H	OH	H
5,11-dideoxyTTX (*)	OH	CH3	H	H	OH	H
5,6,11-trideoxyTTX (*)	H	CH_3_	H	H	OH	H
8-epi-5,6,11-trideoxyTTX	H	CH_3_	H	H	H	OH
**G**	**R1**	**R2**				
4,9-anhydro-5,6,11-trideoxyTTX	H	OH				
4.9-anhydro-8-epi-5,6,11-trideoxyTTX	OH	H				
**H**	**R1**	**R2**	**R3**	**R4**	**R5**	
1-hydroxy-8-epi-5,6,11-trideoxyTTX	OH	H	OH	OH	H	
4-epi-5,6,11-trideoxyTTX	H	OH	H	H	OH	
**I**	**R1**	**R2**	**R3**			
4,4a-anhydro-5,6,11-trideoxyTTX	H	OH	H			
1-hydroxy-4,4a-anhydro-8-epi-5,5,11-trideooxyTTX	OH	H	OH			

**Table 2:** Chemical abstract numbers (CAS) and relative toxicity of TTX analogs [58,59].

TTX Analogs	TEF	CAS Number
TTX	1.0	4368-28-9
11-oxoTTX	0.75	123665-88-3
11-deoxyTTX	0.14	-
11-norTTX-6(R)-ol	0.17	-
11-norTTX-6(S)-ol	0.19	-
4-*epi*TTX	0.16	98242-82-1
4,9-anhydroTTX	0.02	13072-89-4
6,11-dideoxyTTX	0.02	-
5-deoxyTTX	0.01	-
5,6,11-trideoxyTTX	0.01	-
4-CysTTX	-	-
5,11-dideoxyTTX	-	-

* TEF—toxic equivalency factor.

**Table 3:** Characteristic symptoms of TTX human poisoning modified from Noguchi and Ebesu (2001) [66].

Level	Affected System	Specific Symptoms
1	Neuromuscular	Paresthesia of lips, tongue, and pharynx, taste disturbance, dizziness, headache, diaphoresis, pupillary constriction
	Gastrointestinal	Salivation, hypersalivation, nausea, vomiting, hyperemesis, hematemesis, hypermotility, diarrhea, abdominal pain
2	Neuromuscular	Advanced general paresthesia, paralysis of phalanges and extremities, pupillary dilatation, reflex changes
3	Neuromuscular	Dysarthria, dysphagia, aphagia, lethargy, incoordination, ataxia, floating sensation, cranial nerve palsies, muscular fasciculation
	Cardiovascular/pulmonary	Hypotension or hypertension, vasomotor blockade, cardiac arrhythmias, atrioventricular node conduction abnormalities, cyanosis, pallor, dyspnea
	Dermatologic	Exfoliative dermatitis, petechiae, and blistering
4	Respiratory failure, impaired mental faculties, extreme hypotension, seizures, loss of deep tendon and spinal reflexes	

**Table 4:** TTX detection methods, their limits of quantification (LOQs), limits of detection (LODs), and toxicity equivalency factors (TEFs) according to the European Food Safety Authority (EFSA). MBA—mouse bioassay; FLD—fluorescence detection; RB—receptor-based; LC—liquid chromatography; MS—mass spectrometry; HPLC—high-performance liquid chromatography; UVD—ultraviolet detection; SPR—surface plasmon resonance; TLC—thin-layer chromatography; GC—gas chromatography.

Analysis Method	LOD	LOQ
MBA [12,36,52,89]	1.1 μg·g^−1^ [89]	-
Enzymatic assays [31,36,52,73,77,82,89,91,92,93]	2 ng·mL^−1^ [92]	-
TLC–MS [13,72]	0.1 μg [72]	-
HPLC–FLD [84,94,95]	1.27 μg·g^−1^ [94]	
GC–MS [76,84,95]	0.5 μg·g^−1^ [76]	1.0 μg·g^−1^ [76]
LC–MS/MS/UPLC–MS/MS [33,40,96,97,98]	0.09–16 ng·mL^−1^ [33,40,96,97,98]	5–63 ng·mL^−1^ [40]
SPR [30]	0.3–20 ng·mL^−1^ [30]	-
HPLC–FLD [15,32,99]	40-100 ng·g^−1^ [15]	-

**Table 5:** The incidence of TTXs in the Indian Ocean. NPI—no poisoning incidents, MBA—mouse bioassay; FLD—fluorescence detection; LC—liquid chromatography; MS—mass spectrometry; HPLC—high-performance liquid chromatography; UVD—ultraviolet detection; TLC—thin-layer chromatography; GC—gas chromatography.

Producing Species	Vector	Sample Tissue	Location	Country	Poisoning Date	TTX	Detection	Maximum Concentration	Poisoning Victims	Reference
**Australia**										
Unknown	Puffer fish *Lagocephalus scleratus*		Close to Fremantle Hospital	Australia	13 May 1996	TTX	Symptomatology	-	3 people	[23]
Unknown	Puffer fish *Lagocephalus scleratus*		Port Hedland	Australia	1998	TTX	Symptomatology	-	1 person	[24]
Unknown	Toad fish *Tetractenos hamiltoni*		New South Wales	Australia	1 January 2001 to 13 April 2002	TTX	Symptomatology	-	11 people	[100]
Unknown	Toad fish *Tetractenos hamiltoni*	Urine		Australia	2004	TTX	HPLC–UVD	5 ng/mL	7 people	[80]
		Serum						20 ng/mL		
**Asian countries**										
Unknown	Puffer fish		Khulna	Bangladesh	April 18 2002	TTX	Symptomatology	-	45 people	[27]
Unknown	Puffer fish *Takifugu oblongus*	Skin	Khulna	Bangladesh	18 May 2002	TTX	MBA	18.9 MU/g	36 people, 7 deaths	[16]
		Muscle						4.4 MU		
		Liver						4.9 MU/g		
		Gonads						132.0 MU/g		
		Viscera categories						37.0 MU/g		
			Natore					-		
			Dhaka							
Unknown	Puffer fish	Liver	Khulna	Bangladesh	24 July 2005	TTX	Symptomatology	-	6 people	[22]
Unknown		Skin	Khulna	Bangladesh	25 March 2006	TTX	LC–MS/MS	25.35 μg·g^−1^	NPI	[33]
						Anhydro		7.71 μg·g^−1^		
						11-Deoxy		1.12 μg·g^−1^		
						Trideoxy		15.31 μg·g^−1^		
		Muscle				TTX		1.64 μg·g^−1^		
						Anhydro		-		
						11-Deoxy		-		
						Trideoxy		-		
		Liver				TTX		45.71 μg·g^−1^		
						Anhydro		29.17 μg·g^−1^		
						11-Deoxy		-		
						Trideoxy		9.09 μg·g^−1^		
		Ovary				TTX		356.00 μg·g^−1^		
						Anhydro		85.87 μg·g^−1^		
						11-Deoxy		26.00 μg·g^−1^		
						Trideoxy		2,929.70 μg·g^−1^		
Unknown	Puffer fish		Dhaka	Bangladesh	2008	TTX	Symptomatology	-	11 people	[25]
Unknown	Puffer Fish		Narshingdi	Bangladesh	April and June 2008	TTX	Symptomatology	-	95 people, 14 deaths	[26]
			Natore							
			Dhaka							
Unknown	Puffer Fish		Dhaka City	Bangladesh	October 2014	TTX	Symptomatology	-	11 people, 4 deaths	[18]
Unknown	Puffer fish	-	Khulna	Bangladesh	-	TTX	Symptomatology	-	37 people, 8 deaths	[28]
Unknown	Puffer fish *Chelonodon patoca*	Liver	Bay of Bengal	India	June 1998 to March 2001	TTX	MBA	25.9 MU/g	NPI	[61]
		Ovary						183 MU/g		
	*Sphaeroides oblongus*	Liver						16 MU/g		
		Ovary						7.9 MU/g		
	*Lagocephalus inermis*	Liver						5.5 MU/g		
		Ovary						28.9 MU/g		
	*Lagocephalus lunaris*	Liver						5.9 MU/g		
		Ovary						16.6 MU/g		
Unknown	Puffer fish *Chelenodon potoca*	Liver	Bengal coast	India	June 2000–March 2001	TTX	MBA	27.8 MU/g	NPI	[35]
		Ovary						156.7 MU/g		
	*Takifugu oblongus*	Liver						11.75 MU/g		
		Ovary						29.1 MU/g		
	*Lagocephalus lunaris*	Liver						9 MU/g		
		Ovary						30.1 MU/g		
	*Lagocephalus inermis*	Liver						5.7 MU/g		
		Ovary						9.64 MU/g		
*Kytococcus sedentarius*	Puffer fish *Arothron hispidus*	Skin	Annankil fish landings at Parangipettai	India	2010	TTX	MBA	-	NPI	[65]
		Intestine						-		
		Liver						-		
*Cellulomonas fimi*		Muscle						4.4 MU		
		Liver						4.9 MU/g		
		Gonads						132.0 MU/g		
*Bacillus lentimorbus*		Viscera categories						37.0 MU/g		
			Natore					-		
			Dhaka					-		
Unknown	Puffer fish *Arothron stellatus*	Muscles	Parangipettai	India	2016	TTX	HPLC–FLD, TLC–UVD	Qualitative	NPI	[64]
		Gonads				4-*epi*				
		Liver				anhydro				
Unknown	Puffer fish *Takifugu oblongus*	Skin	Kasimedu fishing harbor, Chennai, Tamil Nadu	India	2016	TTX	MBA	75.88 MU/g	NPI	[62]
							GC–MS	16.5 MU/g		
							HPLC	18 MU/g		
		Liver					MBA	143.33 MU/g		
							GC–MS	32.5 MU/g		
							HPLC	48 MU/g		
		Ovary					MBA	163 MU/g		
							GC–MS	34.5 μg		
							HPLC	51 μg		
Unknown	Puffer fish	-	Johor	Malaysia	May 2008	TTX	Symptomatology	-	34 people	[68]
Unknown	*Carcinoscorpius rotundicauda*	Urine	Kota Marudu	Malaysia	June–August 2011	TTX	GC–MS	1.3–602 ng/mL	30 people	[88]
Unknown	Puffer fish *Xenopterus naritus*	Muscle	Manggut	Malaysia	February and July 2013	TTX	LC–MS/MS	27.19 μg/g	NPI	[63]
			Kaong					16.09 μg/g		
Unknown	Puffer fish *Lageocephalus scitalleratus*		Alexandra Hospital	Singapore	2013	TTX	Symptomatology		1 person	[20]
Unknown	*Tetraodon nigroviridis*	Reproduc tive tissue	Satun	Thailand	April to July 2010	TTX	LC–MS/MS, MBA	63.57 MU/g	NPI	[36]
		Liver						97.08 MU/g		
		Digestive tissue						43.33 MU/g		
		Muscle						22.12 MU/g		
	*Arothron reticularis*	Reproductive tissue						-		
		Liver						2.08 MU/g		
		Digestive tissue						3.16 MU/g		
		Muscle						4.02 MU/g		
**African countries**										
Unknown	Puffer fish *Lagocephalus lunaris*	Gonads	National Research Center, Dokki, Cairo,	Egypt	September 1990 through May 1991	TTX	TLC–UVD, MBA	752 MU/g	NPI	[34]
		Liver						246 MU/g		
		Muscles						127 MU/g		
		Digestive tract						221 MU/g		
		Skin						119 MU/g		
Unknown	Puffer fish *Lagocephalus sceleratus*	Gonads	Attaka fishing harbor	Egypt	October 2002 and June 2003	TTX	MBA	3950 MU/g	NPI	[37]
Unknown	Puffer fish *Lagocephulus scleratus*	Muscle	Suez Gulf	Egypt	23 December 2004	TTX			7 people	[21]
Unknown	Puffer fish		Nosy Be Island	Madagascar	July 1998	TTX	MBA	16 UM/g	3 people, 1 death	[19]
Unknown	Puffer fish *Lagocephalus sceleratus*	Liver	Reunion Island	Reunion Island	10 September 2013	TTX	MBA, LC–MS/MS	95 MU/g	10 people	[29]
		Flesh						5 MU/g		
Unknown	Puffer fish, *Tetraodontidae family*		Zanzibar	Tanzania		TTX	Symptomatology	-	1 death	[17]
